# Two Complementary Approaches toward Geodetic Monitoring of a Historic Wooden Church to Inspect Its Static and Dynamic Behavior

**DOI:** 10.3390/s23208392

**Published:** 2023-10-11

**Authors:** Zdzisław Mikołaj Pawlak, Ireneusz Wyczałek, Piotr Marciniak

**Affiliations:** 1 Institute of Structural Analysis, Poznan University of Technology, 60-965 Poznań, Poland; zdzislaw.pawlak@put.poznan.pl; 2 Faculty of Civil and Environmental Engineering and Architecture, Bydgoszcz University of Science and Technology, 85-796 Bydgoszcz, Poland; 3 Institute of Architecture, Urban Planning and Heritage Protection, Poznan University of Technology, 60-965 Poznań, Poland; piotr.marciniak@put.poznan.pl

**Keywords:** wooden building, static and dynamic behavior, inclination monitoring, column displacements, data correlation

## Abstract

To support the conservation efforts regarding a wooden church in Domachowo, extensive research and design work was required to strengthen its weakened structure. A variety of data and analyses are necessary to make an accurate assessment, including obtaining a dimensional model, monitoring the behavior of the structure and its response to external forces, and also performing strength analyses and verifying them with measurement data. For accurate geometric evaluation, static and dynamic measurements were required. A mathematical model and a flowchart of the necessary tasks were developed, along with the selection and installation of measuring devices. For this particular structure, static measurements were made using an automatic total station, and dynamic measurements using tilt sensors. The purpose of the analysis was to correlate the inertia measurements with the absolute tachymetric observations related to reference points fixed outside the object in order to accurately assess the behavior of the object. Another important issue was to model the column element under study in such a way that its horizontal displacements could be determined from the measured inclinations. The obtained results indicated the need to strengthen the joints of the two main parts of the structure in order to minimize the impact of dynamic weather conditions. The paper describes the measurement process, the method of calculating displacements, and the correlation of both types of data. Selected results confirming the conclusions are also presented.

## 1. Introduction

Ensuring the operational safety of buildings is a fundamental requirement when allowing people to stay in them. When potential threats have been identified, engineering measures should be taken in order to strengthen the structure of the facility, restrict access or limit its use, or—in extreme cases—exclude it from use altogether. Engineering decisions in this area must always be based on a thorough assessment of the technical condition of the facility and its response to external forces. The key aspect of this assessment is the diagnosis of the condition of existing structures, and geodetic structural monitoring is a tool that can support this diagnosis. Classic geodetic monitoring methods can detect static geometric changes [1,2] with sub-millimeter precision [3], but only in selected points and at specific time intervals [4]. To expand the measurement possibilities, engineering geodesy employs other sensors, such as radar, lidar, microwave sensors (a greater spatial range of measurement) [5] and, more recently, inertial MEMS sensors, which enable a much greater frequency of measurements [6,7,8]. Inclinometers have been used for many years to analyze the behavior of reinforced concrete structures [9,10,11,12], but they have not been used extensively for the analysis of wooden structures, particularly historic buildings.

Through combining classic geodetic methods with inertial sensors, it is possible to integrate various displacement measurement methods and, at the same time, calibrate sensor readings and mutually verify the correctness of both groups of devices [13]. This approach can extend the spatial range of measurements and increase their frequency, thus enabling conclusions to be drawn regarding both the static and the dynamic behavior of the tested object [14,15].

The research problem requiring the use of such an approach appeared in the case of a historic church in Domachowo in central Poland, a wooden building with structural relics dating back to the 14th century, which is an exceptional situation in the case of such type of buildings [16]. Wooden structures were most often built using one of two methods: carcass (log walls overlapping each other) or skeletal, in which the wooden frame is covered with boards and possibly filled with a more or less compact building or insulating material [17]. In the case of the discussed structure, there is a combination of the log walls of its oldest part, stiffened by a mullion and transom skeleton, and the skeletal structure of the remaining, newer parts of the object [18]. This solution can be found in many old wooden buildings [19]. On the basis of the site inspection, a threat to the stability of the structure was identified: the status of the junction of the two types of structures was in doubt. Before making a decision regarding the possible strengthening of the building’s structure, it was decided to monitor, in particular, the post-and-beam skeleton in the main nave and presbytery. Due to the high historical value of the facility and the unknown operating conditions, it was decided to use combined measurement methods, including static and dynamic surveys, which allow for the precise analysis of the structure’s operating condition.

The contents of the paper comprise a short description of the space under study, the modelling of the column deformation, the measurement techniques used, the arrangement of sensors and targets, and the solutions to technical problems for the effective use of results obtained with traditional geodetic methods and with sensors. The obtained results indicate the object’s susceptibility to the dynamic impact of gusts of wind. However, with regard to the methods of correlation for the readings from both measurement methods, it was considered that the corrective methods used for the purposes of these analyses are sufficient, but there is still room for further research in this area. The insights gained can be effectively used in the study of the construction of other wooden objects.

## 2. Test Object and Methodology of Measurement

### 2.1. Motivation

**Figure 1:**
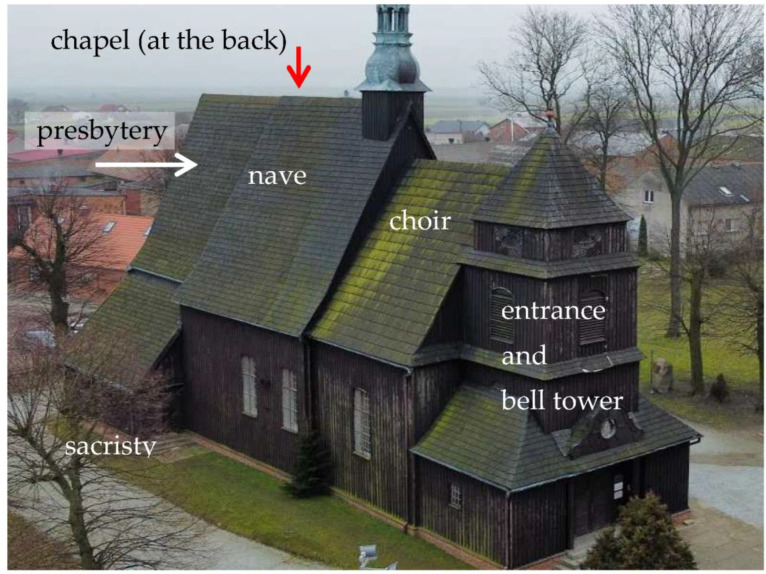
Side view of the tested object—individual parts were created at different times and with different techniques, and their connections reveal the different behavior of the joined parts (source: own photograph).

The examined sacral building consists of several functional parts (Figure 1), erected at different periods and using different techniques, combined with a wealth of changes that have occurred over several centuries of the object’s existence. As a result, discontinuities are visible at the junction of parts created at different times and using different methods. Also noticeable are uneven vibrations in various parts of the object during its operation.

**Figure 2:**
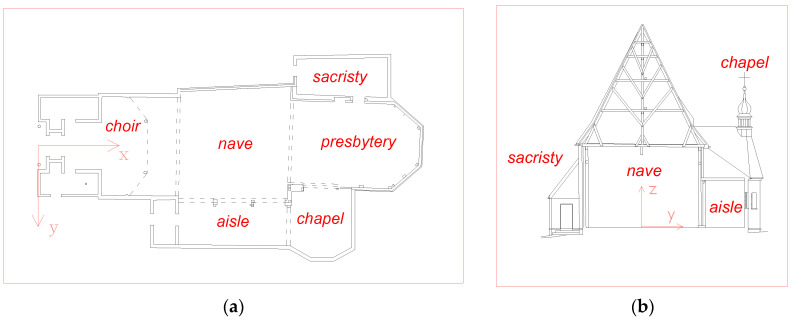
Plan (**a**) and cross-section (**b**) of the body of the given church.

Figure 2 shows the drawings of the body of the analyzed building. The walls of the presbytery are a log structure made from cut oak logs, with a cross-section of about 13 × 50 cm, reinforced with a wooden skeleton.

**Figure 3:**
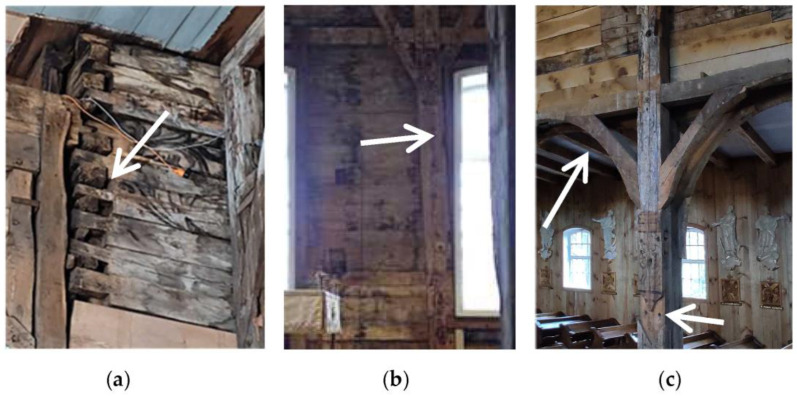
Examples of damage or discontinuity in the wall structure: (**a**) wear to the log construction joints, (**b**) inclination of the frame structure beams in relation to the (newer) windows, (**c**) removed or cut struts reinforcing the frame structure (source: own photographs).

The inner surfaces are partly covered with medieval iconography and ornaments [16]. Due to their age, the ends of the logs at the junction of the walls show clear signs of degradation (Figure 3a).The walls of the nave are a simply supported frame structure with boarding, which was rebuilt over time, weakening it—the inclination of the beams is visible (Figure 3b), as well as the disassembly of a part of the structure at the junction of the nave and the aisle (Figure 3c) [18].

In buildings consisting of parts created at different times, using different methods, weak spots usually occur at the junction of different types of construction, at the point where they are connected [20]. It is expected that the most sensitive element of the building will be the intersection of the two oldest parts, the nave and the presbytery and, in particular, the connection between the presbytery and the main nave and the chapel and the aisle in the southeastern corner of the nave.

### 2.2. Mathematical Model for Measuring the Movements of the Wall of a Wooden Building

Old building structures are generally stable, but this does not preclude slow tilting, subsidence, or a combination of both. In the case of impulsive external forces (e.g., gusts of wind), it had been noticed that the structure reacts with forced vibrations. Thanks to the internal damping of the structure, after the pressure is released, the vibrations disappear and, finally, the structure returns to its initial state [18]. It can, therefore, be assumed that, as a result, there are two types of phenomena: static, consisting of slow changes in the foundation and inclination; and dynamic, expressed in the form of vibrations in response to external pressure. While static displacements are best measured using geodetic instruments, this equipment cannot keep up with the measurement of the fast-changing phenomena characterizing the dynamics of the object. In the latter case, it is reasonable to use tilt sensors, e.g., inclinometers, tilt meters, or electronic levels. The use of both measurement methods requires the determination of the relationship between the inclination and the displacement of individual elements (points) of the tested object. Secondly, it is necessary to bring the measurement results to a common frame of reference.

A mathematical model of the deformation of a column, wall, or any near-vertical element in an existing building requires certain initial assumptions. First of all, it is necessary to determine whether the lower node of the element can rotate freely (hinge) or is blocked from rotation (fixed). The second assumption concerns the flexibility of the analyzed bar. Therefore, the mathematical model of the test element enforces the number of necessary measurement points and the minimum number of inclinometers used per element.

#### 2.2.1. One Inclinometer

**Figure 4:**
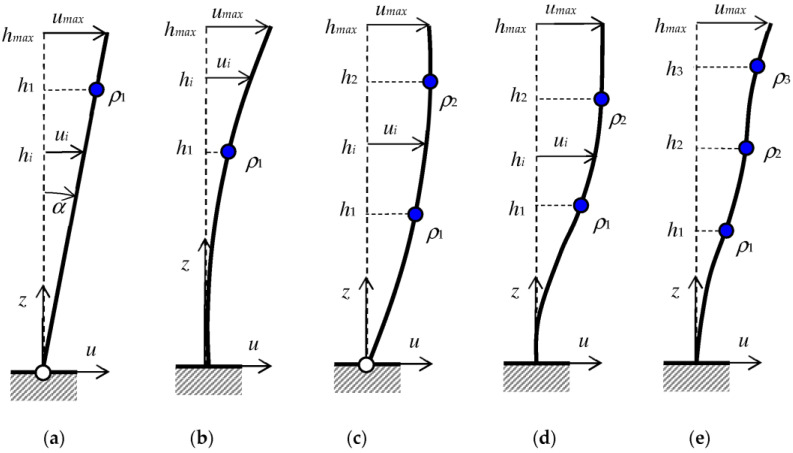
Illustration of assumptions for modelling column deflection lines with different bottom supports: (**a**) hinged, linear function, (**b**) fixed, quadratic function, (**c**) hinged, quadratic function, (**d**) fixed, cubic function, (**e**) fixed, higher-degree polynomial. The blue circle is the measuring point (inclinometer).

In the simplest case, it can be assumed that the bottom support is a hinge, and the whole element rotates around the lower node as a rigid body (Figure 4a). The origin of the coordinate system is taken at the bottom node, the *z*-axis is directed upward along the element, and the horizontal displacements $u\left(z\right)$ are determined. In this case (model H1P), the displacements u(z) are described by a linear function:

(1) $$u\left(z\right)=a_{1}\cdot z=\tan\alpha \cdot h$$

where $a_{1}=\tan\alpha$ is the directional coefficient of the linear function, α is the angle of inclination of the element, and z=h is the height or the level for which the displacement is determined (Figure 4a). A single inclinometer measurement is sufficient to determine the constant $a_{1}$. Therefore, in order to combine absolute and inertial measurements, it is possible to embed the tilt sensor in any place in the beam, and the tachymetric measuring target at the required height above the floor (i.e., $z=h_{1}$).

Assuming that the beam is rigid, and the displacement vector $u_{1}$ at height $h_{1}$ is known, the displacement $u_{i}$ at any level $h_{i}$ can be calculated from the relation:

(2) $$u_{i}=\frac{u_{1}}{h_{1}}\cdot h_{i}$$

In particular, at the top of the rigid beam ($h_{max}$), the displacement will have a maximum value $u_{max}$.

For a rigidly fixed beam at the lower node, deformation is only possible if the element behaves elastically (Figure 4b), meaning that the deflection line has a curvature determined by a higher-degree function. In this case (model F1P), the deflection has to be modeled by a curve described by a polynomial of at least the second degree:

(3) $$u\left(z\right)=a_{2}\cdot z^{2}+b_{2}\cdot z+c_{2}$$

where $a_{2}$ and $b_{2}$ are coefficients whose values should be determined. As the function representing the section rotation angle $\rho \left(z\right)=\tan(\alpha (z))$ is the derivative of the displacement function $u\left(z\right)$, then:

(4) $$\rho \left(z\right)=du\left(z\right)/dz=2a_{2}\cdot z+b_{2}$$

It is worth noting that, in the case of small angles, $\rho \left(z\right)≅\tan(\rho (z))$. From the boundary condition, one obtains:

(5) $$u\left(z=0\right)=0\;\to c_{2}=0\;\rho \left(z=0\right)=0\;\to b_{2}=0$$

which means that a measurement of the rotation $\rho_{1}$ performed with a single inclinometer located at a given height $h_{1}$ is enough to determine the value of the coefficient of the quadratic function:

(6) $$\rho_{1}\left({z=h}_{1}\right)=2\;a_{2}h_{1}\;\to a_{2}=\rho_{1}/2\;h_{1}$$

In turn, the displacement $u_{i}$ of any point located at height $h_{i}$ can be obtained using the formula:

(7) $$u_{i}=\frac{\rho_{1}}{2\;h_{1}}\cdot h_{i}^{2}$$

#### 2.2.2. Two Inclinometers

In the case where the lower node is a hinge and the element deforms elastically, only the first boundary condition from set (5) is valid. To describe the deflection line with quadratic Function (3), we need two inclinometers (Figure 4c) and two measurements of the inclination $\rho_{1}$ and $\rho_{2}$ in radians, at points located at heights $h_{1}$ and $h_{2}$, respectively. In practice, it is necessary to precisely correlate the locations of at least two pairs of targets and inclinometers. Then, the coefficients of quadratic Function (3) have the form (8):

(8) $$a_{2}=\frac{\rho_{1}-\rho_{2}}{2(h_{1}-h_{2})}\;b_{2}=\frac{\rho_{1}h_{2}-\rho_{2}h_{1}}{h_{1}-h_{2}}\;c_{2}=0$$

The displacement $u_{i}$ of any point located at height $h_{i}$ of any hinged-supported element (model H2P) can be calculated from the formula:

(9) $$u_{i}=a_{2}\cdot h_{i}^{2}+b_{2}\cdot h_{i}$$

where the coefficient $a_{2}$ and $b_{2}$ are defined by Equation (8).

Assuming that the lower node is fixed (model F2P), using only two measurement points, the deflection line can be described by a third-degree function (see Figure 4d):

(10) $$u\left(z\right)=a_{3}\cdot z^{3}+b_{3}\cdot z^{2}+c_{3}\cdot z+d_{3}$$

and the inclination, or angle of rotation of the cross-section can be represented by a second-degree polynomial as the derivative of Function (10):

(11) $$\rho \left(z\right)={3a}_{3}\cdot z^{2}+2b_{3}\cdot z+c_{3}$$

Using the two boundary conditions at the bottom node, the two coefficients of the above polynomials can be determined:

(12) $$u\left(z=0\right)=0\;\to d_{3}=0\;\rho \left(z=0\right)=0\;\to c_{3}=0$$

and with two inclination measurements $\rho_{1}\left({z=h}_{1}\right)$ and $\rho_{2}\left({z=h}_{2}\right)$, the other two coefficients can be calculated as:

(13) $$a_{3}=\frac{\rho_{1}h_{2}-\rho_{2}h_{1}}{3h_{1}h_{2}(h_{1}-h_{2})}\;b_{3}=\frac{-\left(\rho_{1}h_{2}^{2}-\rho_{2}h_{1}^{2}\right)}{2h_{1}h_{2}(h_{1}-h_{2})}$$

Finally, the displacement $u_{i}$ of any point located at the height $h_{i}$ of the element rigidly supported at the bottom (Figure 4d) can be calculated from the expression:

(14) $$u_{i}=a_{3}\cdot h_{i}^{3}+b_{3}\cdot h_{i}^{2}$$

where the coefficient $a_{3}$ and $b_{3}$ are defined by Equation (13).

#### 2.2.3. Required Number of Inclinometers

Summarizing the above considerations, it can be concluded that the horizontal deflection function of any vertical element can be described by a polynomial of any degree (e.g., *n*). Assuming that one node is rigidly fixed, then the number of measurement points (inclinometers) one less than the degree of the polynomial (i.e., *n* − 1) is needed to determine the deflection function of this element. On the other hand, when the rotation of the support node is arbitrary or unknown, the same number of points at which the inclination is measured is needed as the degree of the polynomial adopted to describe the deflection (i.e., *n*).

All the above models assume the existence of a single beam, which is practically not reflected in spatial constructions. In each case, different inclinations can be expected along the wall of the object, and different ones across it. The former should assume values close to zero due to the high stiffness of the wall in its plane, while the latter should illustrate the reaction on forces perpendicular to the wall plane, e.g., the wind pressure on the object. It can be assumed that the complex structure of the wall causes the mutual constraint of individual structural elements, which is irrelevant in the case of movement in the transverse direction and, to a much lesser extent, reflects the interconnections of individual structural elements. The last statement is not always true, but it is sufficient to describe the object’s reaction to external pressure. In timber frame constructions, the columns are usually placed freely on the ground. However, they are connected with screws, formwork, and the possible filling of the wall plane. This limits the movement of the column in relation to the free model, but should not significantly distort the model of transverse inclination to the wall surface, especially in the simply supported variant.

A knowledge of the relationship between inclinations and displacements in the absolute system allows the definition of the relationships between the inclinometer readings and the tachymetric measurement.

### 2.3. Comparison of Results Obtained Using Different Mathematical Models

**Figure 5:**
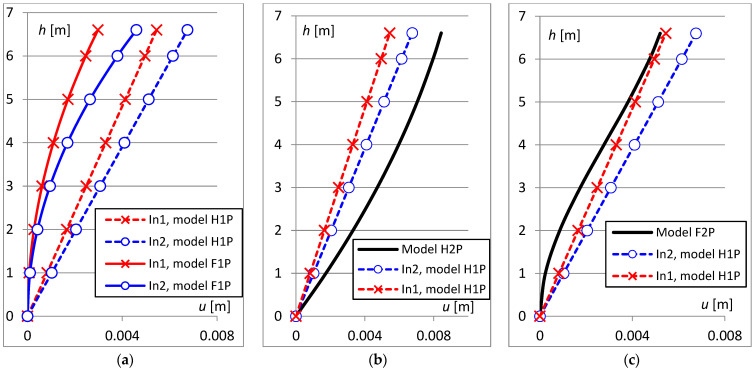
Deformation diagrams of the analyzed column determined for different models: (**a**) hinge and linear functions (dash line), fixed with the quadratic function (solid line), (**b**) hinge with linear (dash line) and quadratic functions (solid line), (**c**) hinge and linear functions (dash line), fixed with the cubic function (solid line).

**Figure 6:**
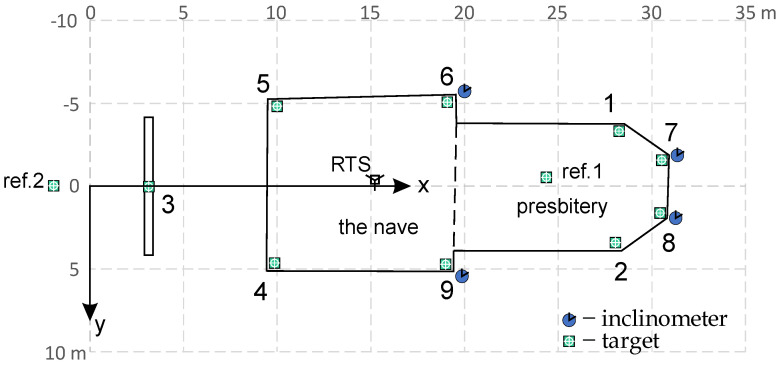
Scheme of the body of the church, with the location of the main components of the monitoring system—the robotic total station (RTS), targets (green squares), and inclinometers (blue circles).

The results of the inclinometer measurements were used to determine the deflection line and the maximum displacement of the top of the selected column (see Figure 5). Depending on which mathematical model was chosen for the analysis, the shape of the deflection line was different but, more importantly, different values for the maximum horizontal displacement were also obtained. Measurements from two inclinometers attached to the selected column were used for the analysis: the first (In1), marked as number 9 in Figure 6, located at a height of $h_{1}=6.05\;m$; and the second (In2) below, at a height of $h_{2}=4.85\;m$. And the maximum displacement $u_{max}$ was determined at the top of the column, at a height of $h_{max}=6.80\;m$. The tilt values measured on a windy day at these points were:

(15) $$\rho_{1}\left(h_{1}=6.05\right)=0.0473{}^{\circ}=0.0008257\;\left[rad\right]\;\rho_{2}\left(h_{2}=4.85\right)=0.05863{}^{\circ}=0.0010232\;\left[rad\right]$$

In the case of the H1P model and the F1P model, measurements from only one device are sufficient to determine the deflection line. Figure 5a shows a comparison of the results obtained when only the first inclinometer was used for the analysis (crosses on the graph) with the results when only the second inclinometer was used (circles on the graph). After the adoption of a linear deformation model for the column (model H1P) and hinge, larger displacement values were obtained than for curvilinear deformation and stiffened support (model F1P).

In the next analysis, measurements from both inclinometers were used to determine the curvilinear deformation of the second degree, with the hinge at the bottom (H2P model). The curvilinear deformation from the H2P model was also compared with the H1P straight-line models but, in this case, larger displacement values were obtained (Figure 5b).

Finally, an analysis was performed in which measurements from both inclinometers were used, and the deformation was described by a third degree curve, with the lower node fixed (F2P model). In this case, displacement values were obtained that were closest to straight-line deformations (Figure 5c). In addition, it was found that a single inclinometer placed at the top of the column allows the estimation of the maximum displacements in the straight-line model (H1P) similar to the curvilinear model (F2P), where measurements from two inclinometers are needed.

The maximum displacement values determined at the top of the column for the models considered are summarized in Table 1. Based on the analysis, it can be concluded that, in the case of monitoring a historical object, when the maximum displacement values need to be determined, the best solution is to place one inclinometer in each column, but at the top of it.

### 2.4. Measurements

In order to check the technical conditions of, and the extent of deformations to, individual parts of the structure of the monitored building, and their reaction to external factors, the following actions were taken [18]:

- an orthogonal coordinate system (grid) was defined, with the *x*-axis directed along the church, from the center of the entrance door to the middle of the rear wall of the presbytery, and the *y*-axis directed to the right, in accordance with the geodetic definition of the grid (see Figure 6),
- for static measurements (steady state of the object), the tachymetric method was selected with the use of the automated Total Station Leica TCRP 1201+, with the 1″ precision of angle measurement and 2 mm precision of measured distances, and targets made from reflective foil (for distances of about 6 m, this precision gives the standard error RMSE for determining a horizontal displacement not greater than 0.05 mm),
- for dynamic measurements (vibrations), the electronic inclinometer BWsensing WF/WM series 400 was used, with an inclination measurement accuracy of 0.005 ± 0.001° (for a wall about 7 m high, this corresponds to linear values of 0.60 mm ± 0.12 mm, where the second component is time-variable).

Using the much more accurate tachymetric method, it was possible to correct the inclinometer results, which are less accurate due to the occurrence of possible time-varying systematic errors.

The arrangement of the target points and sensors is shown in Figure 6. The targets are marked with numbers from 1 to 9, and the common places of targets and inclinometers are numbers 6, 7, 8 and 9. Static measurements were made in 1-month time intervals, which enabled the tracking of construction movements, and also the periodic control of the expected drift of the inclinometer indications.

### 2.5. Preliminary Interpretation of the Measurement Results of a Given Object

**Figure 7:**
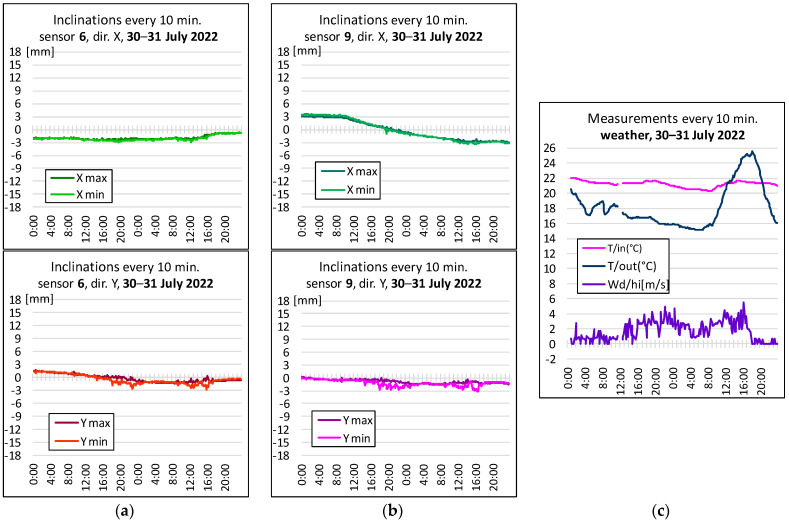
Inclinations for calm weather on 30–31 July 2022: (**a**) in point 6, (**b**) in point 9, in the longitudinal direction x (at the top), in the transverse direction y (at the bottom), (**c**) the temperature inside and outside the building (15 ÷ 25 °C) and wind gusts (0 ÷ 5.2 m/s).

**Figure 8:**
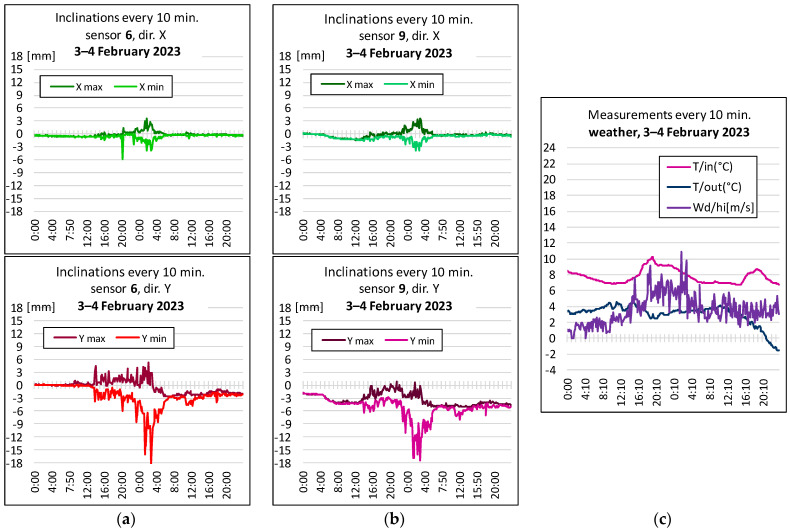
Inclinations for windy weather on 3–4 February 2023: (**a**) in point 6, (**b**) in point 9, in the longitudinal direction x (at the top), in the transverse direction y (at the bottom), (**c**) the temperature inside (7.4 ÷ 10.2 °C) and outside the building (−2.0 ÷ 5.3 °C), and wind gusts (0 ÷ 11.0 m/s).

In the case of the tested object, the corners of the nave near the presbytery, i.e., in the vicinity of points 6 and 9 (shown in Figure 6), were considered the weakest places. Both targets and inclinometers were installed there; additionally, inclinations were measured at two corner points on the rear wall of the chancel (points 7 and 8). According to the theoretical assumptions, BWsensing WF/WM 400 sensors were used to measure the inclinations, while the static displacements were measured using the Leica TCRP 1201+ total station, and weather conditions using the Sencor SWS 12500 WiFi weather station located near the facility, on its northwestern side, at a height of about 5 m. Assessing that the tested beams are not anchored to the foundations (hinges) and are characterized by a high stiffness, inclination measurements were made with one inclinometer (variant H1P) and converted to linear values for the floor level (i.e., 6.8 m). The obtained values are recorded on weekly charts. To illustrate some of these, Figure 7 and Figure 8 show two graphs of two-day oscillations (with lines showing the minimum and maximum values) along the *x*- and *y*-axes, as well as the values for the wind strength and temperature inside and outside the facility. Figure 7 shows the state of the building in stable weather conditions; it shows smooth changes in the inclination along the *x*-axis and slight vibrations in the y-direction resulting from the operation of the building.

Figure 8 illustrates the building’s response to gusts of wind. The correspondence of the inclinations with the readings for the wind gusts is visible (see Figure 7c and Figure 8c), confirming the validity of the assumptions made earlier. An important problem is the shift of the graphs (inclination) in relation to the initial indications, which could suggest slow changes in the inclination of the tested beams. Due to the stability of the object confirmed via the tachymetric measurement, it was assumed that this is the effect of the drift of the sensors used, so the amount of vibration of the object was determined each time in relation to the average readings for windless weather in a given month.

Based on the observation that, of all external factors, strong gusts of wind have the greatest influence on the structure, it is necessary to correlate the indications of the anemometer with the measured inclination. The dynamic nature of weather phenomena combined with the delayed reaction of the structure does not allow for a mathematical relationship between the two factors with a high frequency, so it was decided that they would be compared in 10 min blocks, based on (instantaneous) wind readings of gust strength and direction, and readings of extreme slope components, respectively, x min, x max, and y min, y max. For this purpose, the graphs illustrate the relationship between gusts of wind (as well as the temperature inside the building and outside, in its vicinity) and the inclination of the structure in selected locations. Atmospheric phenomena monitored by a weather station located near the object are also recorded in a 10 min cycle, thanks to which it measures, over the same time, the values of the same factors that affect the object.

The general correlation coefficient between the wind gust forces and slope magnitudes for the most vulnerable point No. 9 was calculated, according to the known formula:

(16) $$corr\left(w_{n},u_{n}\right)=\frac{\sum_{i=1}^{n}\left(w_{i}-{\bar{w}}_{n}\right)\left(u_{i}-{\bar{u}}_{n}\right)}{\sqrt{\sum_{i=1}^{n}{\left(w_{i}-{\bar{w}}_{n}\right)}^{2}}\sqrt{\sum_{i=1}^{n}{\left(u_{i}-{\bar{u}}_{n}\right)}^{2}}}=\frac{cov\left(w,u\right)}{\sigma_{w}\sigma_{u}}$$

where:

$w_{i}$, ${\bar{w}}_{n}$—the *i*-th value of the slope of the column and the average slope at the level of the ceiling [mm],
$u_{i}$, ${\bar{u}}_{n}$—the *i*-th gust of wind and the average value of the gust [m/s],
$\sigma_{w},\;\sigma_{u}$—the standard deviation values for the slope and for the gust.

The closer to unity (i.e., 1.0) the value of $corr\left(w_{n},u_{n}\right)$, the greater the correlation between the considered parameters. At node 9 (point No. 9), discussed here, a correlation coefficient of about 0.7 was obtained via comparing a one-day set of wind forces and slope readings from the two sensors, measured on a windy day.

As shown in Figure 8, during strong wind, the vibrations occur in both directions, although, according to theoretical considerations, they are greater along the *y*-axis. After the wind stops, there is stabilization, but residual shifts of points (column inclination) of up to about 5 mm remain. However, this is not a permanent trend because, over time, there is a return to the initial state, or a deviation of a different nature arises, although in the same range of a few millimeters.

### 2.6. The Problem of Compliance of the Inclinometer Indications with the Results of the Static Measurement

**Figure 9:**
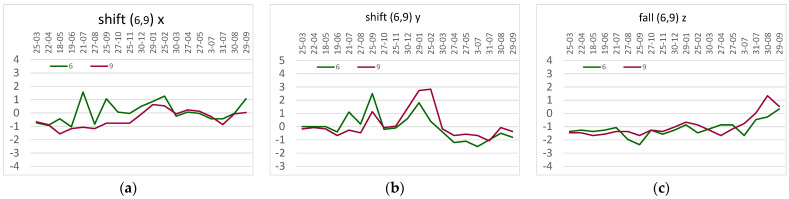
Diagrams of horizontal displacements in 19 measurement series at points 6 and 9 most susceptible to vibrations: (**a**) along the church—*x*-axis, (**b**) across the church—*y*-axis, (**c**) vertically—z-axis [mm].

In order to correct the sensor indications throughout the test period, static measurements with a total station were made at 1-month intervals on quiet mornings, when there should be no vibrations in the tested structure. In this way, the components of the inclination of the measured points in the directions of the axes of the adopted coordinate system (and the component of height) were determined. Due to the analyses discussed here, the values at points 6 and 9 were the most important; the results obtained for both points in a given time interval are shown in Figure 9. The graphs below show that the displacements along the *x*- and *z*-axes do not exceed 3 mm, and that those along the *y*-axis do not exceed 4 mm but, most importantly, they do not show a significant trend of change.

Taking into account the standard errors in the tachymetric measurement, it was initially assumed that the object at the level of the ceiling does not show unambiguous horizontal displacements relative to the floor. Meanwhile, the inclinometer readings were characterized by a variability of a few to several millimeters even at rest and were different for different sensors. This observation is consistent with the specifications of the devices used and confirmed by the results of similar studies using inertial sensors [18].

The heterogeneity of changes in readings suggests that they do not depend on the influence of external factors (identical measurement conditions throughout the facility), but only on the specificity of the sensor’s operation; they are also not linearly variable.

Two actions have been taken to correct the phenomenon of senor drift:

1. the correction of indications using second-degree polynomial equations as a function of time (in a 1-month step of readings),
2. the simultaneous measurement of the inclination of the same element with two sensors.

The first approach was discussed in a paper at the FIG Congress in Warsaw [18], indicating that, thanks to the second-degree correction, it is possible to “zero” the readings in a quiet time. It was also found that, for the description of the examined phenomenon of forced vibrations, the absolute values of the indications are not important, but their variability, as a result of the impulse pressure of external forces, is [21].

The second corrective action was performed in the last phase of the research by moving sensor no. 7 to the structural element measured with sensor no. 9, but at a lower height (about 4.8 m). In further work, the upper point 9 was marked as In1, and the lower one as In2, in accordance with theoretical analyses. The angular values of the inclinations were, each time, converted into displacements under the ceiling (at the level of 6.8 m). The indications of both sensors were compared for a calm period, which was characterized by a small influence of the wind (unfortunately, no violent weather phenomena were recorded at that time). It turned out that both sensors show slightly different inclination values, which suggests that the tested beam does not meet the assumed stiffness condition, or the sensor indications are not sufficiently stable. Measurements taken with two inclinometers placed on a single column were further analyzed using mathematical models that describe the curvature of the element during its deformation.

### 2.7. Selected Final Results after Corrections of Inclinometers

**Figure 10:**
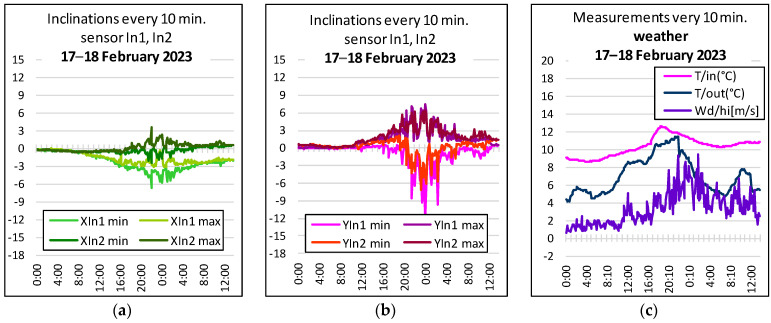
Summary of the inclination values of the part of the structure in the most vulnerable location (point 9) at levels 4.6 (In2) and 6.05 m (In1): (**a**) the inclination along the *x*-axis, (**b**) the inclination along the *y*-axis, and (**c**) the readings for the wind force (0.2 ÷ 8.6 m/s) and temperature, in a cumulative 10 min cycle.

In order to illustrate the obtained results, Figure 10 show graphs of displacements of the same column measured by sensors In1 and In2, combining them with the indications of atmospheric conditions. Only once during the period under review—on the night of 17–18 February 2023—were significant wind gusts recorded.

The obtained results indicate a significant susceptibility of the tested structure of the nave to the impact of gusts of wind in the direction of the *y*-axis. During the measurements on this very windy evening, the maximum displacement values of −12 mm were found at point 9 at the level of the ceiling (Figure 10b). The results showing vibrations at different levels of the tested column differ slightly. This proves that the phenomenon of vibrations and, thus, horizontal displacements, is more complicated than it could initially be assumed. This, in turn, signals that, when designing a method of strengthening the discussed connection of walls, the object should be controlled at its various levels, while care is also taken of the ongoing control and correction of inclinometer readings.

## 3. Discussion

From a geodetic point of view, the assessment of the results obtained is primarily concerned with ensuring that the intended accuracy is achieved. In this respect, it is difficult to refer to the bibliography, as there are no such records in the available literature on the subject. It was, therefore, assumed that the values obtained would be consistent with the a priori accuracies declared by the manufacturer. The evaluation of the tachymetric measurements consisted of obtaining redundant observations and using typical least-squares-based network alignment algorithms. One of the groups of parameters of equalized quantities is the assessment of RSM errors after adjustment. It was found that the results obtained each time met these accuracy requirements.

With regard to inclinometers, the existence of time-varying systematic errors (bias) signaled by their manufacturer was confirmed, and they were, therefore, corrected based on displacement values obtained from periodic tachymetric measurements. In principle, the ‘floating’ of the inclination readings is irrelevant, because the purpose of these measurements was to determine temporary vibrations as a result of the response to dynamic forces. Therefore, the purpose of the correction was only to bring the inclination values for quiet time closer to the tachymetric measurement results.

The deformation of the building structure can also be affected by other weather parameters, such as the uneven heating of the structure due to sunlight, moisture from rain, snow load, etc. The analysis of the tested facility as a whole confirmed the initial theoretical assumptions that the internal elements are not directly subject to weather factors, and all such loads are carried by appropriately strengthened roof slopes. There were no short-term displacements observed due to the influence of the above factors. In some cases, the inclinometer readings shifted, as shown in Figure 10, and then reversed in a relatively short period of time. The tachymetric measurements were responsible for the long-term (static) assessment of the structure, and they confirmed that there was no tendency for the inclination of the structure to increase over time (see Figure 9). Perhaps, this would be noticeable over a very long period of time, which can be the subject of a separate study.

Summarizing the results of the geodetic measurements obtained, there was no danger to the structure due to static deformation and, in order to mitigate the observed dynamic behavior (excessive vibration), it was decided to design the strengthening of the weakest, short sections of the walls at the junction of the nave and chancel with steel ties. They will be introduced in subsequent stages of conservation and renovation work on the analyzed object. Surveying will continue and will be the basis for evaluating the effectiveness of the strengthening work undertaken.

## 4. Conclusions and Further Work

The basis for assessing the geometric condition of historic objects is a precise description of the shape, based on geodetic measurements, calculations of the static model and, if necessary, testing the reaction of a given object to stress factors. The subject of this publication was the last of the mentioned groups of studies. The tests concern a specific object, but they can be universal for a given group of old wooden buildings, because they present developed approaches to the design, course, and evaluation of the results of combined geodetic measurements (tachymetry) and readings of inclination sensors, which were analyzed together with the obtained values of the selected weather parameters [18,21]. The approach presented here is utilitarian in nature, due to the physical subject and purpose of the research, but it can be a permanent element of monitoring complex structures. The combination of these methods brings the following benefits:

1. It provides the results of the classical long-term (static) monitoring of the horizontal and vertical displacements of the object at its critical points;
2. It monitors vibrations—horizontal displacements caused by the dynamic impact of atmospheric factors, mainly gusts of wind;
3. It enables the calibration of inertial sensors based on the results of static measurements;
4. It associates the size of dynamic displacements with the measured values of the weather parameters that cause them;
5. It illustrates the variability of displacements of individual fragments of the same structural element, which enables a more complete understanding of the nature of the response of the object at a given place to the external factors;
6. It allows the determination of the curvature of the deformed element under load, which makes it possible to estimate the method of support (free rotation or elastic clamping).

For the purpose of this practical application, four inclinometers were used at locations that were initially considered to require special attention. The sensors used for this purpose require a power supply, which slightly complicates the procedure of mounting and replacing the sensors. Installing wirelessly powered models would allow for their more flexible arrangement, resulting from the need to change their seating and, in the case of a larger number of them, to obtain a better image of the phenomena occurring in the tested structure. In the presented research, the results obtained were sufficient for engineering conclusions and construction decisions. An interesting, and still not fully understood, issue is the clear differentiation of the measurement results in different locations; this should encourage more advanced modeling research. As a research topic in the next stage of work, it is planned to monitor the selected column with several sensors located at different levels along its height in order to determine its curvature during vibrations.

On the other hand, from the point of view of surveying, it is essential to meet certain accuracy criteria, including control over the possible occurrence of outliers, a reduction in systematic errors, and the assessment of the level of uncertainty of the results. The necessary procedures have been applied in this respect. The results of multiple, repeatable measurements have shown that the accuracy of the static measurement to be met under these conditions does not exceed 0.5 mm for each component of displacement and, for inclinometers, the error is not greater than 1.0 mm at the top of the beam along each axis. The proposed procedure has proven to be effective and sufficient for monitoring the structure of a historic wooden building and should, therefore, be developed and improved.

## Figures and Tables

**Table 1:** The maximum displacement $u_{max}$ determined for the considered models at the top of the column ($h_{max}=6.80\;m$).

Model	Polynomial Degree	Bottom Support	Position and Number of Required Inclinometers	Maximum Displacement $u_{max}\;[mm]$
H1P	1	hinge	In1	5.45
H1P	1	hinge	In2	6.75
F1P	2	fixed	In1	2.97
F1P	2	fixed	In2	4.60
H2P	2	hinge	In1 and In2	8.44
F2P	3	fixed	In1 and In2	5.20

In Table 1, In1 corresponds to the inclinometer at $h_{1}=6.05\;m$, and In2 corresponds to the inclinometer at $h_{2}=4.85\;m$.

## Data Availability

The detailed measurement results contain data from eighteen months of operation. All data are stored on the servers of the Poznań University of Technology. Due to the protection of information and internal procedures, they may be made available via e-mail contact: ireneusz.wyczalek@pbs.edu.pl.
